# Official Call for a State Medical Convention

**Published:** 1850-01

**Authors:** 


### ARTICLE III.

### OFFICIAL CALL FOR A STATE MEDICAL CONVENTION.

Office of the Secretary, of the
Ottawa Medico-Chirurgical Association.
18th December, A. D., 1849.

*Editors North Western Medical and Surgical Journal.*

GENTLEMEN:—At a regular meeting of the "Ottawa Medico-Chirurgical Association," held at the rooms thereof, on the evening of Monday, the 17th inst., Dr. Edward A. Guilbert offered the following preamble and resolutions, viz:

*Whereas*, it has been deemed advisable by sundry medical gentlemen, that an *official* call for a Convention of Physicians to assemble at Springfield, Ill., at some future time, for the purpose of forming a State Academy of Medicine, should be immediately made: and *whereas*, it is esteemed very proper for those who have originated the movement, to make said *call*, Now therefore, be it

*Resolved*, That the "Ottawa Medico-Chirurgical Association" respectfully, and earnestly recommend that a State Medical Convention, convene at the Capital of the State, on the *first Tuesday* in June, A. D., 1850, for the purpose of forming a "State Medical Association."

*Resolved*, That this (the Ottawa Medico-Chirurgical) Association, do hereby call such a Convention, and that these preamble and resolutions be signed by the President and Secretary of this body, and transmitted to the "North Western Medical and Surgical Journal," for publication.

On motion of Dr. D. D. Morrison, it was further

*Resolved*, That a Committee of conference, consisting of three members of this Association, be appointed to correspond with the Physicians of Springfield, in reference to the arrangements incident to the convocation of said Convention.

These resolutions were adopted, and Drs. D. D. Morrison, Edward A. Guilbert, and Jonathan Pierson, were appointed the committee proposed in the last resolution.

[Signed,] A. H. HOWLAND, M. D.
*President.*
EDWARD A. GUILBERT, M. D.
*Secretary.*

